# Tracheobronchial Amyloidosis Causing Left Lung Collapse: A Case Report

**DOI:** 10.7759/cureus.71658

**Published:** 2024-10-16

**Authors:** Rayhan Karimi, Arun Adlakha, Rob Thomas

**Affiliations:** 1 Internal Medicine, Edward Via College of Osteopathic Medicine, Spartanburg, USA; 2 Pulmonology, Piedmont Medical Center, Rock Hill, USA; 3 Pathology, Piedmont Medical Center, Rock Hill, USA

**Keywords:** amyloid mass, amyloidosis, amyloid plaque, bronchoscopy, chronic bronchitis, congo red stain, lung collapse, tracheobronchial

## Abstract

Tracheobronchial amyloidosis is a rare condition characterized by the deposition of amyloid proteins in the trachea and bronchi, leading to significant respiratory symptoms such as chronic mucoid, cough, dyspnea, and recurrent respiratory infections. We present the case of a 61-year-old individual who developed tracheobronchial amyloidosis, which poses a diagnostic challenge due to its clinical and radiological resemblance to other pulmonary disorders, including chronic bronchitis. Histologically, tracheobronchial amyloidosis is characterized by the presence of amyloid deposits confirmed by Congo red staining, which shows apple-green birefringence under polarized light. Further confirmation can be obtained through electron microscopy, revealing non-branching fibrils. This report explores the clinical presentation, diagnostic challenges, and management of tracheobronchial amyloidosis. Therapeutic interventions may include bronchoscopic procedures to remove obstructive amyloid deposits and systemic treatments such as chemotherapy or immunotherapy to address the underlying amyloid process, aiming to improve patient outcomes and quality of life.

## Introduction

Amyloidosis is a group of rare diseases characterized by the abnormal deposition of amyloid proteins in various tissues and organs, leading to impaired function and diverse clinical manifestations [1]. These proteins abnormally misfold and form insoluble fibrils that aggregate and disrupt normal cellular architecture. There are several types of amyloidosis, classified based on the precursor protein involved, such as AL (primary) amyloidosis, associated with plasma cell disorders, and AA (secondary) amyloidosis, linked to chronic inflammatory conditions [1]. Symptoms vary widely depending on the organs affected and can include fatigue, weight loss, edema, and organ-specific signs like nephrotic syndrome, cardiomyopathy, or neuropathy [1].

Tracheobronchial amyloidosis (TBA) is a rare disorder characterized by the abnormal deposition of amyloid proteins within the trachea and bronchial walls, leading to respiratory complications such as airway obstruction, chronic cough, dyspnea, and recurrent infections. Histologically, tracheobronchial amyloidosis is identified by the presence of amorphous eosinophilic deposits within the submucosa of the tracheobronchial tree [2]. These deposits are confirmed through Congo red staining, which exhibits a distinctive apple-green birefringence under polarized light [2]. Additionally, electron microscopy reveals non-branching fibrils that are typically 7.5 to 10 nm in diameter, further confirming the diagnosis [2]. The identification of these histological features is crucial for differentiating tracheobronchial amyloidosis from other pulmonary conditions with similar clinical presentations.

Tracheobronchial amyloidosis can often be misdiagnosed as chronic bronchitis due to the overlapping clinical presentations of both conditions. Patients with tracheobronchial amyloidosis frequently exhibit symptoms such as chronic cough, sputum production, wheezing, and dyspnea, which are also hallmark features of chronic bronchitis [3]. This symptomatic similarity can lead to delays in the correct diagnosis, especially if the initial evaluation focuses solely on clinical symptoms without further investigation. Radiological imaging and pulmonary function tests may not definitively distinguish between the two conditions [3]. Recognizing these diagnostic differences is vital to avoid misdiagnosis and ensure appropriate treatment, as the management strategies for tracheobronchial amyloidosis differ significantly from those for chronic bronchitis.

Treatment options for tracheobronchial amyloidosis are primarily focused on alleviating symptoms and managing airway obstruction. Bronchoscopic interventions, such as laser therapy, cryotherapy, and mechanical debulking, are commonly employed to remove obstructive amyloid deposits and improve airway patency [4]. In severe cases, stent placement may be considered to maintain airway patency [4]. Systemic treatments depend on the underlying cause and type of amyloid protein involved. For localized amyloidosis, regular monitoring and symptomatic treatment may suffice. In cases associated with systemic amyloidosis, therapies such as chemotherapy, immunotherapy, or targeted agents like bortezomib and daratumumab may be utilized to reduce amyloid production [4].

## Case presentation

We present a 61-year-old male who complained of chronic mucoid productive cough, dyspnea, wheezing, and chest tightness for the last several years. He coughed up mucoid sputum on a daily basis and had been treated on many occasions with antibiotics for intermittent episodes of purulent changes in his sputum. He stated that he had been diagnosed with acute purulent bronchitis on the majority of these occasions, and on others, he was diagnosed with pneumonia. He had not been hospitalized for episodes of pneumonia, and no sputum had been cultured. He had a past medical history of acid reflux, a 25+ year smoking history, nonspecific environmental allergies, anxiety, type 2 diabetes mellitus, hyperlipidemia, hypertension, and sleep apnea and was evaluated for chronic bronchitis and pneumonia. He denied chest pain, fever, hemoptysis, sweating, or weight loss on presentation. There was no significant history of ongoing post-nasal drainage, sinus infections, or any other upper airway issues, needing regular therapy. Additionally, there was no history of exposure to pulmonary tuberculosis or travel outside the state.

Physical examination revealed audible wheezing and episodes of productive cough with purulent sputum. No hemoptysis was noted. He was afebrile with stable vital signs, except for mild tachypnea. His resting respiratory rate was 25 breaths per minute with resting room air oxygen saturation of 96%. Chest examination revealed scattered bilateral rhonchi and crackles overlying the right mid-chest and back.

**Figure 1:**
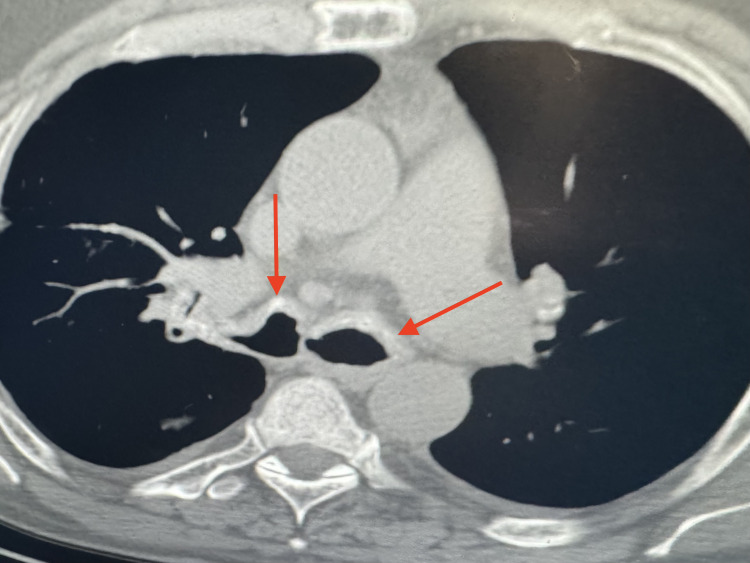
Axial view of chest showing irregular thickening with calcification of the tracheobronchial airways (arrows).

**Figure 2:**
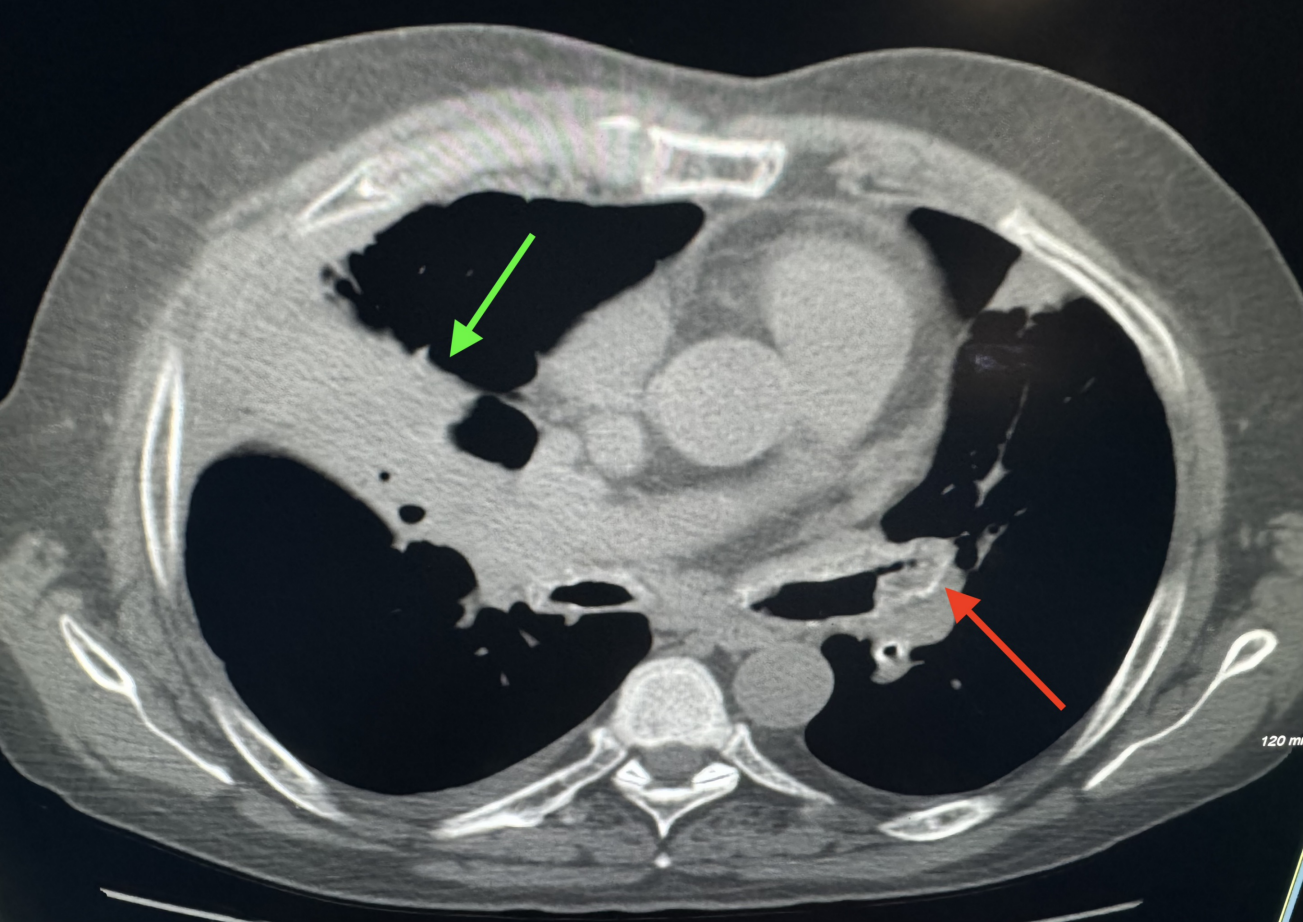
Axial view of chest showing thickening, calcification, narrowing, and occlusion of the left bronchial tree (red arrow). Also seen is airspace consolidation of the right middle lobe of lung (green arrow).

His lab tests revealed mild leukocytosis with dominant neutrophilia and mild hyperglycemia. Baseline sputum and blood cultures were sent which were negative. CT scan of the chest revealed irregular thickening with calcification of bilateral tracheobronchial airways (Figure 1). Also seen was thickening, calcification, narrowing, and occlusion of the left bronchial tree and an airspace consolidation of right middle lobe of the lung (Figure 2).

**Figure 3:**
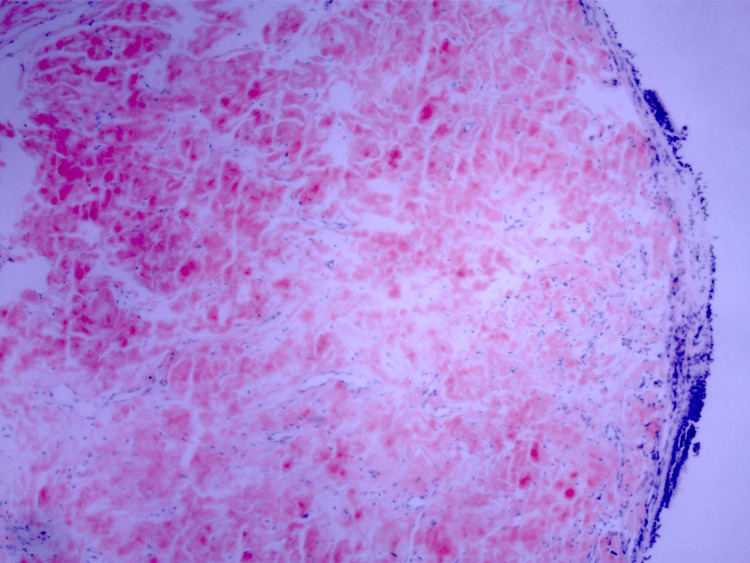
Congo red stained slide showing the red highlighted amyloid deposition beneath the mucosal surface (40x magnification).

There were no nodules, masses, interstitial changes, adenopathy, or pleural disease present. He subsequently underwent diagnostic bronchoscopy which showed diffuse smooth, glistening, translucent, shiny, concentric, inflamed, thickened, and infiltrated tracheobronchial surface. There were no focal nodules, masses, or deposits noted. No bleeding was present. The endobronchial biopsy revealed areas of calcification along with amorphous submucosal proteinaceous material which stained positive with Congo red stain (Figure 3). Pathological findings confirmed the diagnosis of tracheobronchial amyloidosis (TBA).

Subsequent 2D echocardiogram (Echo) and CT scan of abdomen and pelvis were negative for any overt amyloidosis of other organs. He was then treated with IV antibiotics, systemic steroids, bronchodilators, mucolytics, oxygen, and insulin. He responded adequately to the treatment and was discharged home on oral antibiotics, prednisone taper, Mucinex, inhaled corticosteroids, long-acting bronchodilators, and montelukast. He was strongly advised to be evaluated and treated at a medical center for his TBA, including laser therapy. He vehemently refused to go to another medical center for endobronchial laser therapy, other local endobronchial treatments, or systemic therapy.

He continued to see us regularly in our outpatient office. Overall, he remained stable from a respiratory standpoint except for intermittently needing outpatient course of antibiotics to treat his episodes of purulent bronchitis. However, during this time, he did not require hospitalization. His subsequent chest X-rays did not show any worsening changes and therefore was continued on his daily medications.

**Figure 4:**
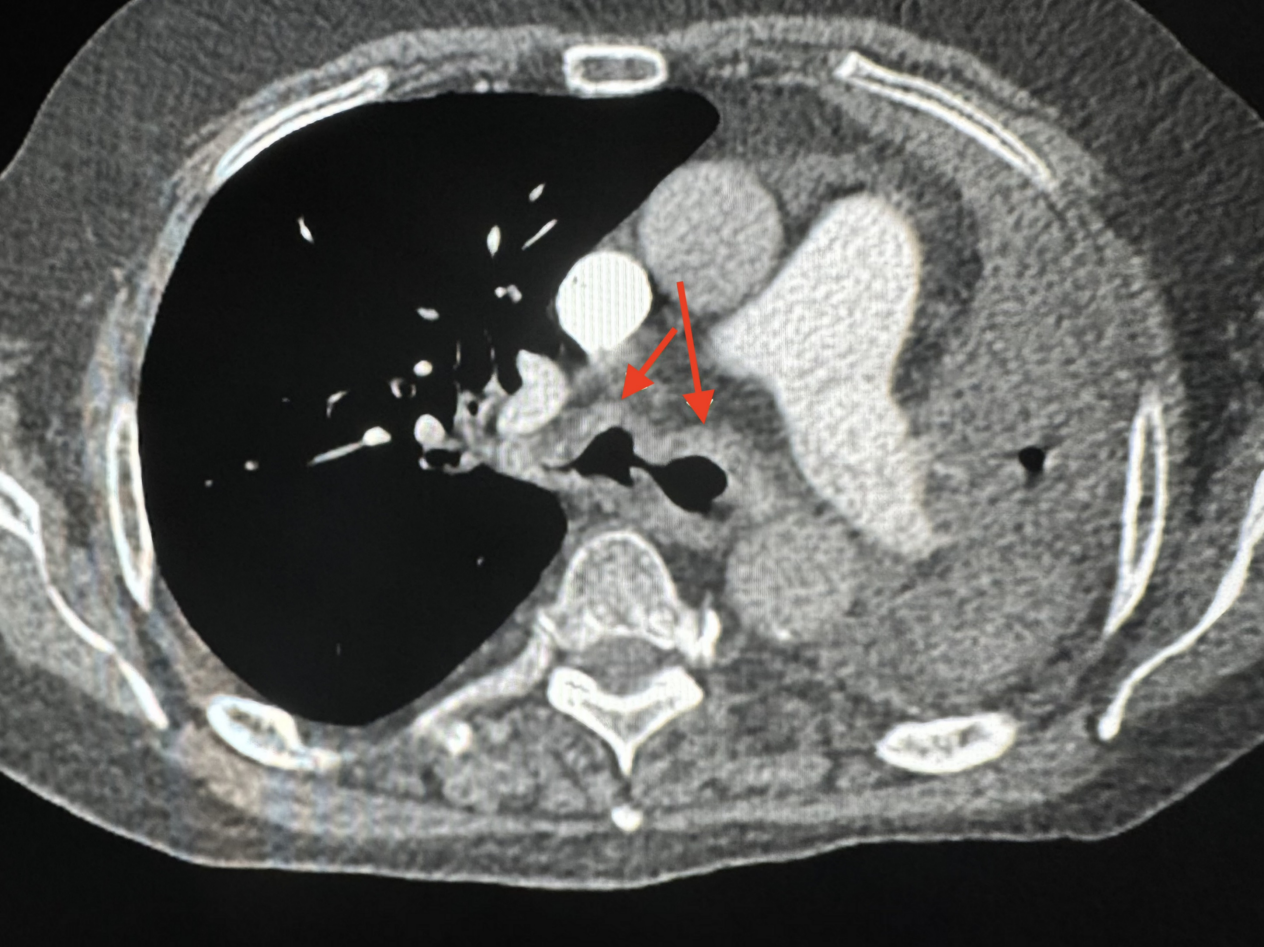
Axial view of chest showing bilateral irregular thickened and calcified airways (red arrows).

**Figure 5:**
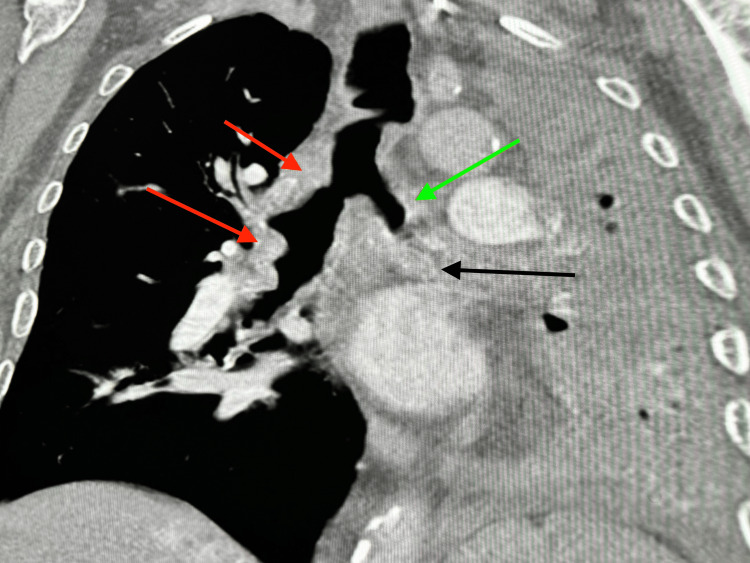
Coronal view of chest showing bilateral irregular thickened and calcified airways (red arrows) with high-grade bronchial narrowing and occlusion of left bronchial tree (green arrow). Also, volume loss and atelectasis of left lung and presence of scattered bronchial calcifications adjacent to the occluded airway were noted (black arrow).

In January 2022, he presented to the emergency room with syncopal spells, severe shortness of breath, and generalized weakness. Clinical examination showed that he was hypoxic at 88%, tachypneic, and in atrial fibrillation. His chest X-ray showed complete opacification of the left hemithorax with some volume loss and mediastinal shift to the left. The right lung field appeared to be clear. He underwent a CT chest which revealed bilateral irregular thickened and calcified airways with high-grade bronchial narrowing and occlusion of the left bronchial tree from thickened and calcified bronchial mucosa. Also, volume loss and atelectasis of the left lung and scattered bronchial calcifications adjacent to the occluded airways were noted (Figures 4, 5).

**Figure 6:**
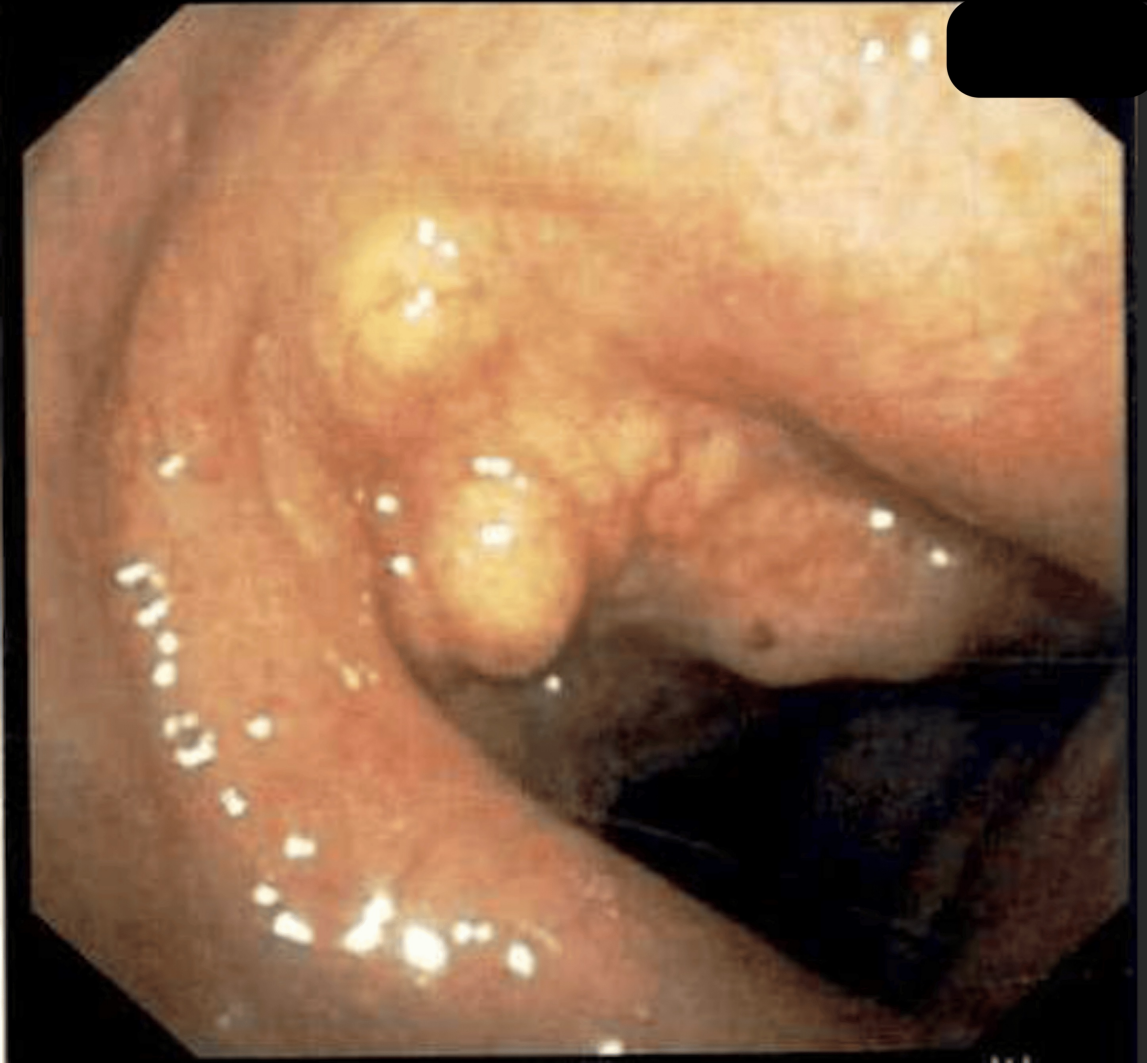
Bronchoscope image showing a cluster of nodular mucosal lesions projecting into the lumen, at the level of the proximal trachea.

He underwent emergent diagnostic and therapeutic bronchoscopy, which revealed multiple nodular and mass-like mucosal endobronchial lesions of the proximal trachea and the bronchial tree, more in the left side than the right. Some of these lesions were projecting into the upper and central airways (Figure 6). Also, infiltrative and occlusive disease with near total occlusion of the left lung from the amyloid lesions was presented.

**Figure 7:**
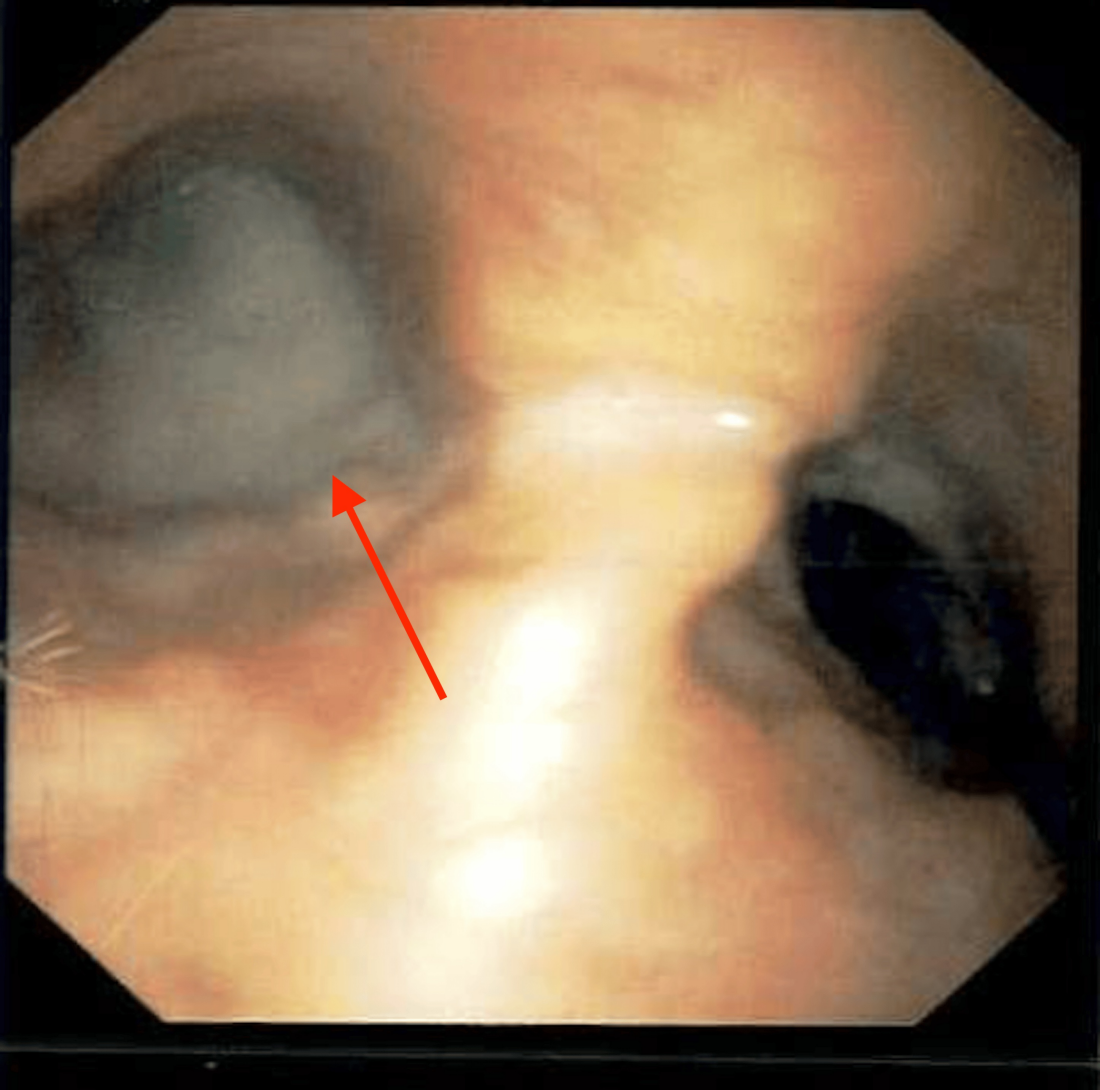
Bronchoscope image showing a thick mucus plug occluding the left main stem bronchus (red arrow).

**Figure 8:**
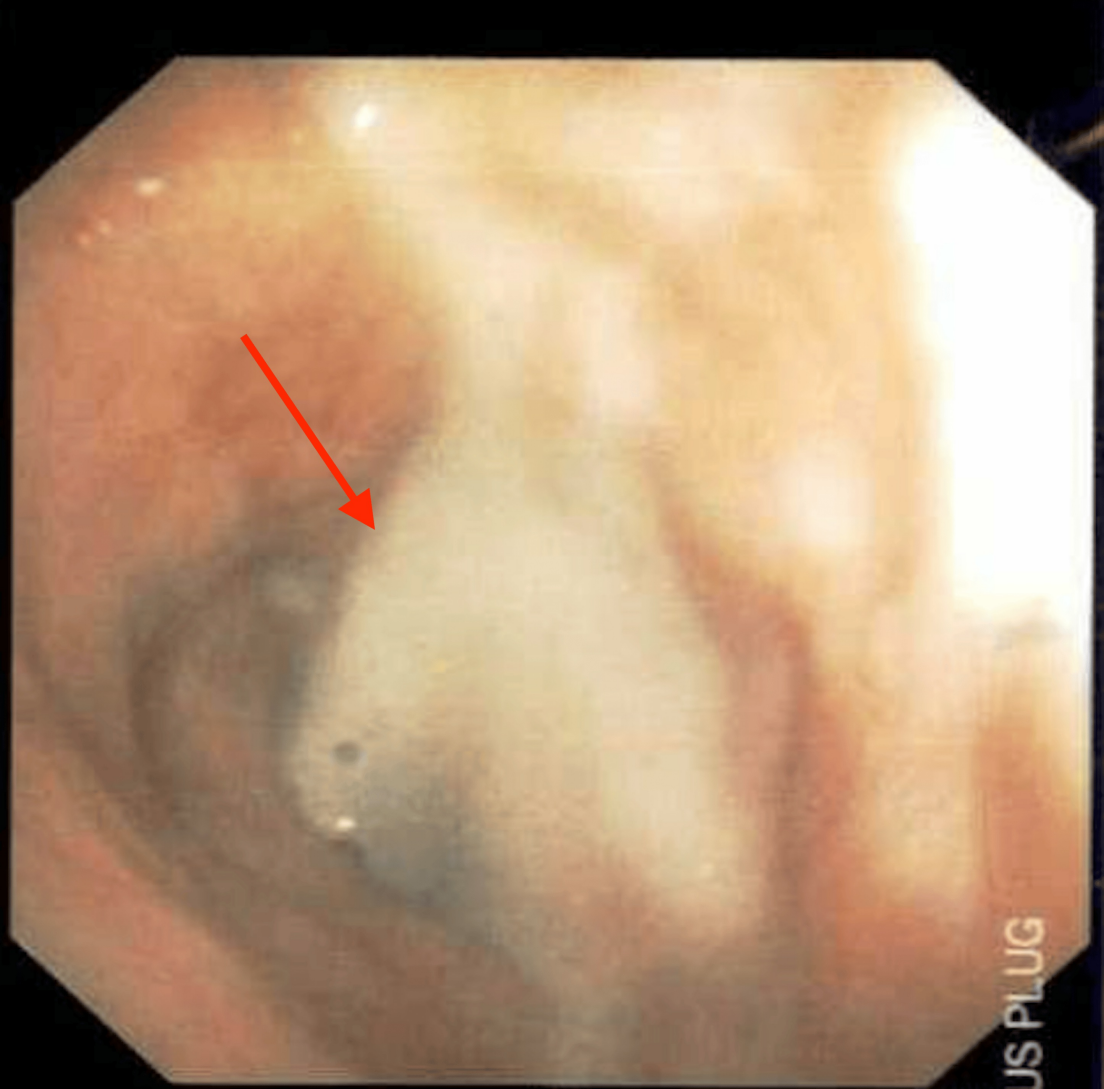
Bronchoscope image showing a mucus plug occluding the left main stem bronchus (red arrow).

**Figure 9:**
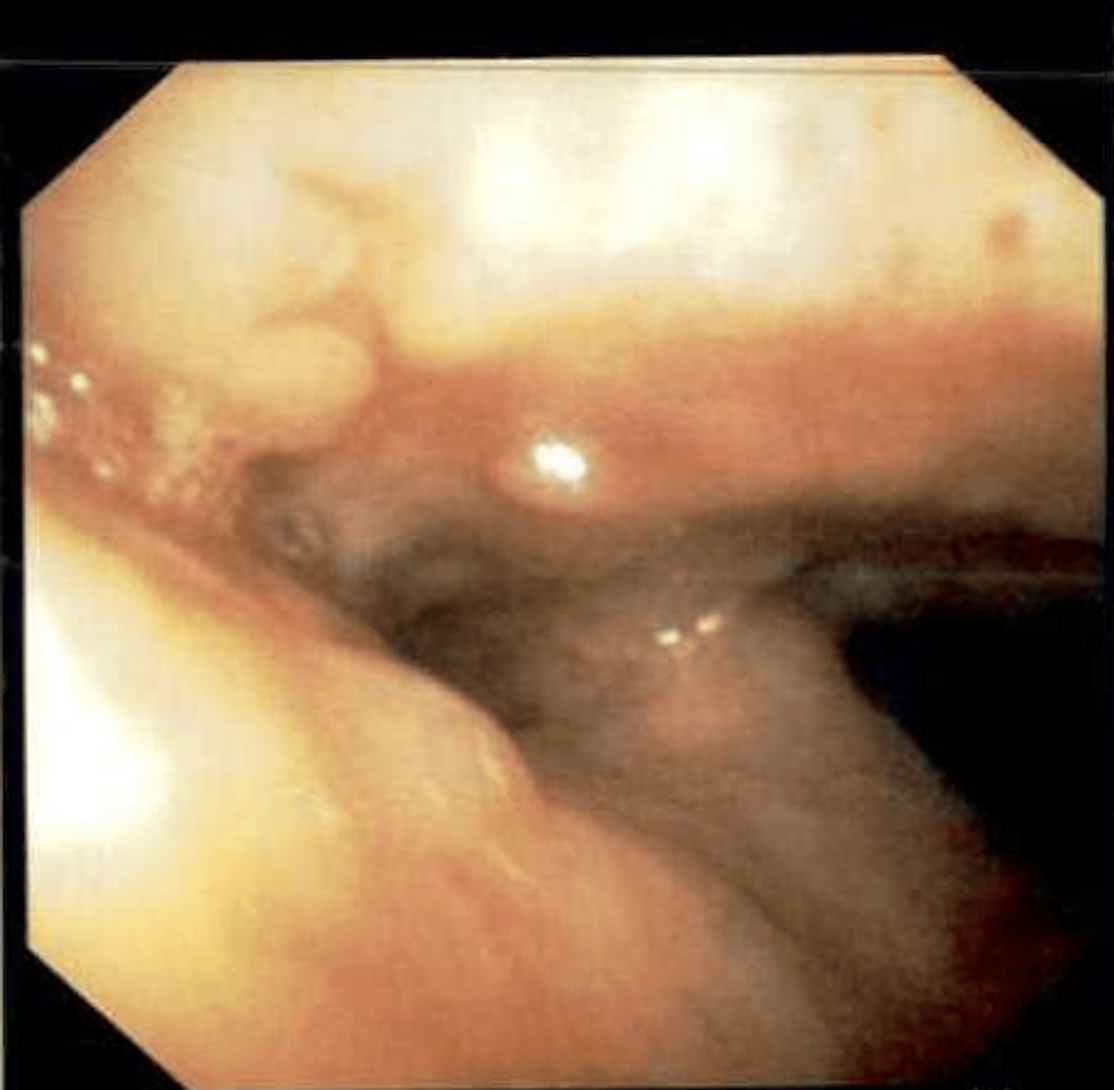
Post-suction bronchoscope image showing amyloid deposits in the distal anterior trachea and anterior carina.

A thick mucus plug was present at the carina and the origin of the left main stem bronchus, which was adequately suctioned and cleared with saline lavage (Figures 7, 8). Post-suction images showed amyloid deposits in the distal anterior trachea and anterior carina (Figure 9).

**Figure 10:**
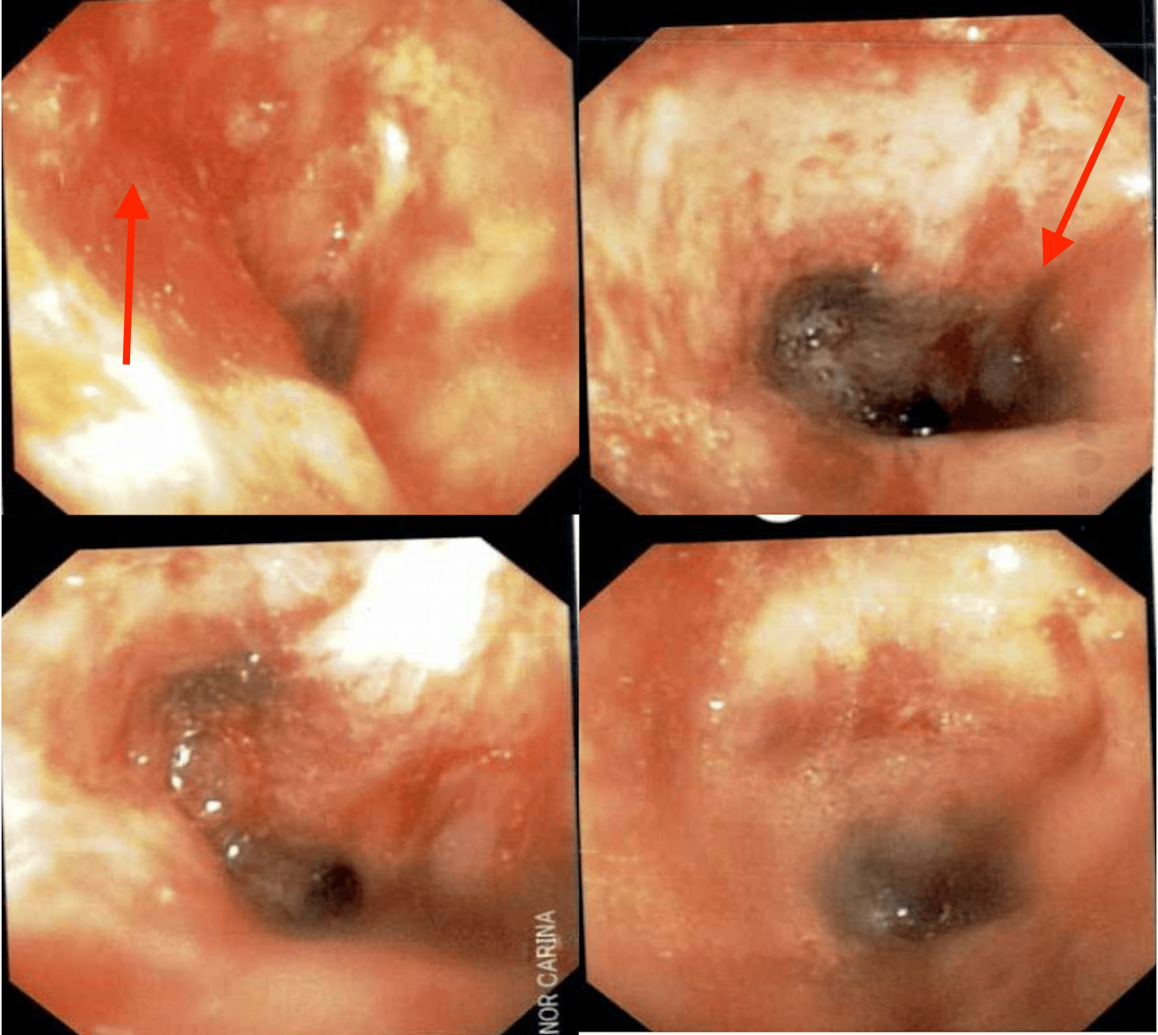
Post-suction bronchoscope images of the left main stem bronchus and minor carina showing constrictive/occlusive disease (left 2 images with arrow) and mucosal nodular disease projecting into the lumen (right 2 images with arrow).

Also, occlusive, constrictive, and nodular disease of the left upper lobe/lingula and mucosal nodular disease projecting into the lumen of the left lower lobe bronchus were noted (Figure 10). Bronchial secretions were sent for cytology and cultures, which were all negative. He was stabilized and discharged in baseline condition.

At this point, he had evidence of both types of TBA, including the circumferential sub-mucosal amyloid and the nodular, mass-like amyloid that was projecting into the lumen of the upper and central airways. The former had led to occlusion and atelectasis of the left lung, and the latter placed him at a high risk for asphyxiation. The infiltrative amyloid lesions could lead to recurrent collapse of the left lung and future collapse of the right lung. Once again, he was strongly advised to be evaluated and treated at a medical center for his TBA, which had progressed over time. He needed multidisciplinary as well as multimodality treatment, such as laser therapy balloon dilation, airway stenting, local steroid/mitomycin injection, cryotherapy, and external beam radiotherapy. He continued to deny any therapy for his TBA lesions.

He currently visits the office infrequently and stays home most of the time. He has not needed another hospitalization for respiratory complications thus far. He is maintained on continuous oxygen, daily low-dose prednisone, as well as all of his other regular medications. On a few occasions, his primary care physician has called in antibiotics to treat episodes of respiratory infections.

## Discussion

Systemic and localized amyloidosis are two distinct forms of amyloidosis, differing primarily in the distribution and impact of amyloid deposits [5]. Systemic amyloidosis involves the widespread deposition of amyloid proteins in multiple organs and tissues, which can lead to severe, multi-organ dysfunction [6]. Common types of systemic amyloidosis include AL (primary) amyloidosis, associated with plasma cell dyscrasias, and AA (secondary) amyloidosis, linked to chronic inflammatory conditions. Symptoms vary widely based on the organs involved and can include renal impairment, cardiomyopathy, neuropathy, and gastrointestinal disturbances [7-9]. In contrast, localized amyloidosis is characterized by amyloid deposits confined to a single organ or tissue, such as the respiratory tract, skin, or urinary bladder. This form often results in localized symptoms specific to the affected area, such as airway obstruction in tracheobronchial amyloidosis or cutaneous lesions in skin amyloidosis. The treatment strategies also differ: systemic amyloidosis typically requires systemic therapies to reduce amyloid production and manage organ dysfunction, while localized amyloidosis often involves localized treatments like laser therapy, surgical excision, or organ-specific interventions [10]. Understanding the distinction between these forms is crucial for accurate diagnosis and effective management.

The cornerstone of the diagnosis of TBA is bronchoscopy with biopsy and positive Congo red staining. CT scans also help in the diagnosis and to detect disease progression. CT often times shows calcifications and thickened or occluded airways [11]. In TBA, there are various presentations that can be observed, including plaques, nodular-type, and constrictive, occlusive lesions [12]. The unique aspect in this case is that the patient began with a plaque type that was causing symptoms resembling chronic bronchitis, which transformed into nodular and occlusive disease. Usually, lesions remain in one form, being nodular or plaque type, respectively. It is observed, through this case, that over time with no treatment, the amyloidosis progresses and begins to infiltrate and occlude the lumen. Additionally, these lesions attract mucus which can plug and cause total occlusion and collapse of a lung, as seen in our case. Therefore, regular monitoring to detect disease progression should be implemented.

Due to the rarity of TBA, there is no established first-line therapy. The treatment of tracheobronchial amyloidosis focuses on relieving airway obstruction and managing symptoms due to the amyloid deposits in the trachea and bronchi. Bronchoscopic interventions are commonly used first, with procedures like laser therapy, cryotherapy, and mechanical debulking to remove amyloid plaques and reopen the airways. These therapies are often highly effective, especially laser debulking. One study showed effective airway opening in 81% of patients treated with laser therapy. However, one drawback to these treatments is the chance of reoccurrence [13]. If tracheobronchial amyloidosis is part of systemic amyloidosis, systemic therapies such as chemotherapy, immunotherapy, or targeted agents like bortezomib and daratumumab are utilized to reduce amyloid production [14]. In our case, the disease was localized; therefore, he was advised to consider laser therapy for treatment; however, he declined all available treatments. In an analysis of 293 cases with localized amyloidosis, a study found that 10.6% of patients exhibited lower respiratory tract involvement. Moreover, there was an 80% progression-free five-year survival rate [15]. With a low risk of progression and a substantial five-year survival rate, a "watch and wait" approach can be considered, as seen in our case. The patient has been living with a collapsed left lung for over five years and symptoms of chronic bronchitis with intermittent respiratory infections. Therefore, our case supports offering patients aggressive treatment to avoid chances of life-threatening complications and for symptomatic relief.

## Conclusions

In conclusion, tracheobronchial amyloidosis is a rare and challenging condition characterized by the deposition of amyloid proteins within the trachea and bronchi, leading to significant respiratory symptoms and potential airway obstruction. This case highlights the importance of accurate diagnosis and the varied treatment approaches available, ranging from bronchoscopic interventions to systemic therapies. Continuous monitoring is crucial to assess for progression to mucosal nodular and constrictive, occlusive disease, which can result in severe respiratory complications and require more aggressive interventions. Ultimately, a multidisciplinary team and personalized management approach are key to optimizing outcomes for patients with tracheobronchial amyloidosis.
